# Supplementary material for: Evidence supported by Mendelian randomization: impact on inflammatory factors in knee osteoarthritis
Source: Front Med (Lausanne). 2024 May 28;11:1382836. doi: 10.3389/fmed.2024.1382836 (PMC11165061; doi:10.3389/fmed.2024.1382836)
Supplement: Supplementary file 1 [file Data_Sheet_1.ZIP › data/GCST90274793_5e6/793.leaveoneout.pdf]

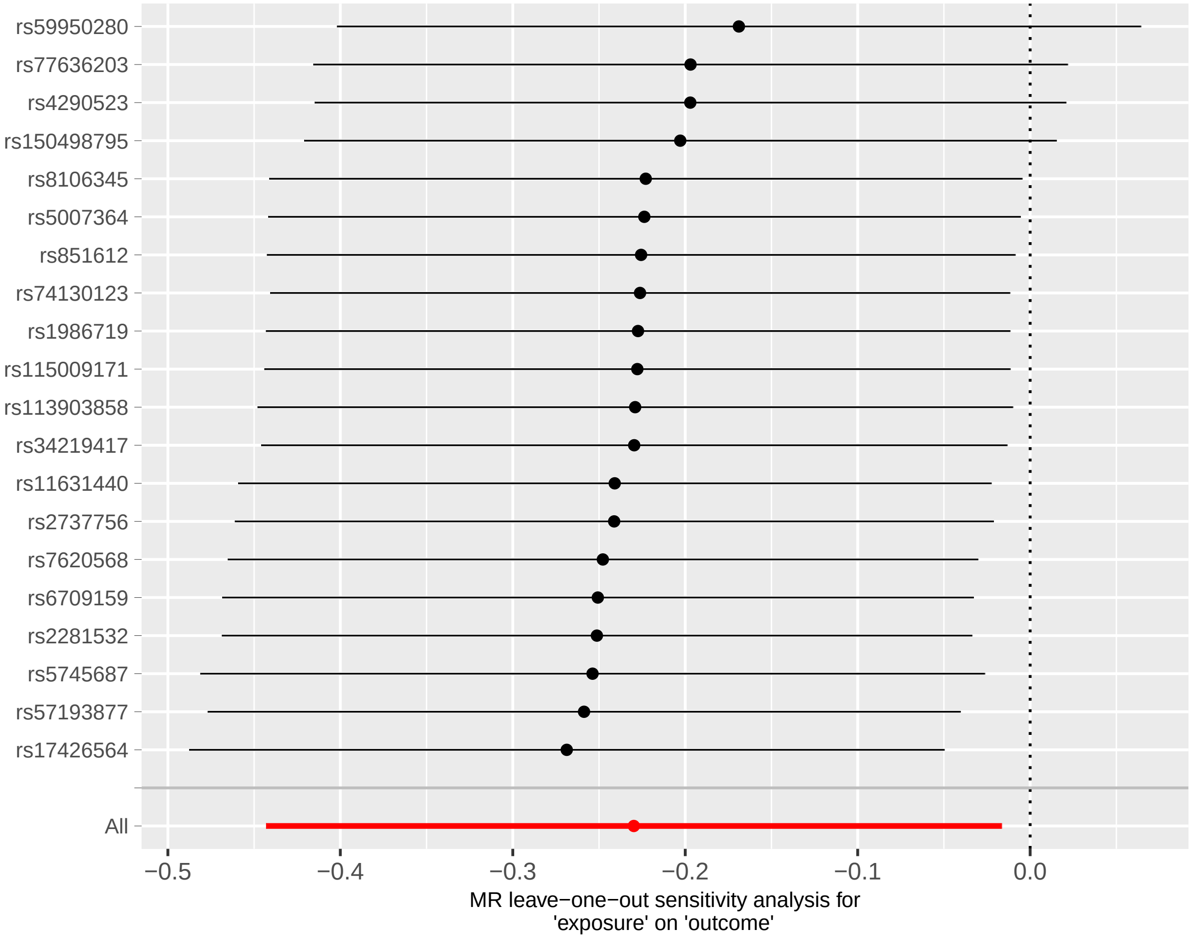
rs59950280
rs77636203
rs4290523
rs150498795
rs8106345
rs5007364
rs851612
rs74130123
rs1986719
rs115009171
rs113903858
rs34219417
rs11631440
rs2737756
rs7620568
rs6709159
rs2281532
rs5745687
rs57193877
rs17426564
All
−0.5
−0.4
−0.3
−0.2
−0.1
0.0
MR leave−one−out sensitivity analysis for
'exposure' on 'outcome'
